# The impact of coal mine dust characteristics on pathways to respiratory harm: investigating the pneumoconiotic potency of coals

**DOI:** 10.1007/s10653-023-01583-y

**Published:** 2023-05-02

**Authors:** Conchita Kamanzi, Megan Becker, Muazzam Jacobs, Petr Konečný, Johanna Von Holdt, Jennifer Broadhurst

**Affiliations:** 1 Department of Chemical Engineering, Minerals to Metals Initiative, University of Cape Town, Cape Town, South Africa; 2 Department of Chemical Engineering, Centre for Minerals Research, University of Cape Town, Cape Town, South Africa; 3 Department of Environmental and Geographical Science, University of Cape Town, Cape Town, South Africa; 4 Division of Immunology, Department of Pathology, Institute for Infectious Diseases and Molecular Medicine, Neuroscience Institute, University of Cape Town, Cape Town, South Africa; 5 National Health Laboratory Service, Johannesburg, South Africa

**Keywords:** Coal workers’ pneumoconiosis, Coal mine dust, Pulmonary toxicity, Dust properties, Pathogenic pathways

## Abstract

Exposure to dust from the mining environment has historically resulted in epidemic levels of mortality and morbidity from pneumoconiotic diseases such as silicosis, coal workers’ pneumoconiosis (CWP), and asbestosis. Studies have shown that CWP remains a critical issue at collieries across the globe, with some countries facing resurgent patterns of the disease and additional pathologies from long-term exposure. Compliance measures to reduce dust exposure rely primarily on the assumption that all “fine” particles are equally toxic irrespective of source or chemical composition. For several ore types, but more specifically coal, such an assumption is not practical due to the complex and highly variable nature of the material. Additionally, several studies have identified possible mechanisms of pathogenesis from the minerals and deleterious metals in coal. The purpose of this review was to provide a reassessment of the perspectives and strategies used to evaluate the pneumoconiotic potency of coal mine dust. Emphasis is on the physicochemical characteristics of coal mine dust such as mineralogy/mineral chemistry, particle shape, size, specific surface area, and free surface area—all of which have been highlighted as contributing factors to the expression of pro-inflammatory responses in the lung. The review also highlights the potential opportunity for more holistic risk characterisation strategies for coal mine dust, which consider the mineralogical and physicochemical aspects of the dust as variables relevant to the current proposed mechanisms for CWP pathogenesis.

## Introduction

Air pollution has been reported as the fourth leading risk factor for mortality worldwide, accounting for approximately 6.7 million deaths in 2019 (HEI and IHME, 2020). Of these deaths, approximately 20% are accounted for by chronic respiratory diseases such as pneumoconiosis (caused by fugitive mineral dust exposure). Historically, epidemic levels of morbidity and mortality from such diseases have been recorded in mining settings, due to the associated chronic exposure of individuals to dust (Patra et al., 2016; Perret et al., 2017; Ross & Murray, 2004).

Mine dust has been linked to a range of pneumoconiotic diseases, where the most prominent examples of this class of dust-induced diseases are silicosis, coal workers’ pneumoconiosis (CWP), and asbestosis. In modern terms, these diseases are commonly thought to be relics of the past, based on the assumption that modern technologies and dust control strategies could manage dust concentrations to safe levels (Cohen, 2016). However, CWP remains a prevalent occupational dust disease across the globe, accounting for 25% of the total pneumoconiosis cases in 2017 (Shi et al., 2020).

Apart from occupational exposure, studies have demonstrated that coal mine dust can disperse to proximal communities (Huertas et al., 2012, 2014; Mishra et al., 2012). As exposure limits for ambient areas are set at lower levels compared to occupational settings, irrespective of whether they are affected by the same source, mining communities may be vulnerable to a range of respiratory diseases. To date, studies have recorded cases of emphysema, chronic obstructive pulmonary disease and bronchitis in individuals proximal to coal mines (Laney & Weissman, 2014; Yadav & Jamal, 2018). However, trends in the global prevalence of CWP suggest that cases of these diseases are greatly underreported for countries that fall into the middle SDI (sociodemographic index), due to disparities in clinical reporting schemes and surveillance programmes (Shi et al., 2020). Thus, apart from posing a serious occupational health hazard, coal mine dust may constitute a significant public health concern for coal-producing countries, particularly in the Global South where established mining communities are prevalent.

By definition, CWP is classified as an interstitial lung disease caused by the inhalation of coal mine dust and the subsequent damage of the lung tissue (Laney & Weissman, 2014). Apart from the well-established link between CWP and coal mine dust, no clear causal agent(s) have been consistently identified for the disease across different global contexts. As coal dust particles compose of varying amounts of carbonaceous (organic) and mineral matter (inorganic), studies have debated and demonstrated the role that minerals play in pathways leading to CWP development (Cohn et al., 2006a; Harrington et al., 2012; Kuempel et al., 2003; Schoonen et al., 2010; Song et al., 2022; Sun et al., 2022; Zosky et al., 2021). In addition to coal mine dust produced from the mined material, coal mine dust in the broader context of potential dust sources can also contain diesel exhaust particle, which have been associated with acute pulmonary damage (Hiura et al., 1999; Mauderly et al., 1987; Maynard & Kuempel, 2005; Stoeger et al., 2006). Despite knowledge of these various pathways to pulmonary damage, mechanisms to screen the factors relating to the pneumoconiotic potency of coal mine dust remain underexplored. To begin to address these concerns, an understanding of the hypotheses/discoveries related to the management of CWP and coal mine dust diseases is needed to describe the shift in understanding that coal mine dust may possess inherent and variable pneumoconiotic potency.

### Historical advancements in the management and mitigation of coal mine dust diseases

**Fig. 1:**
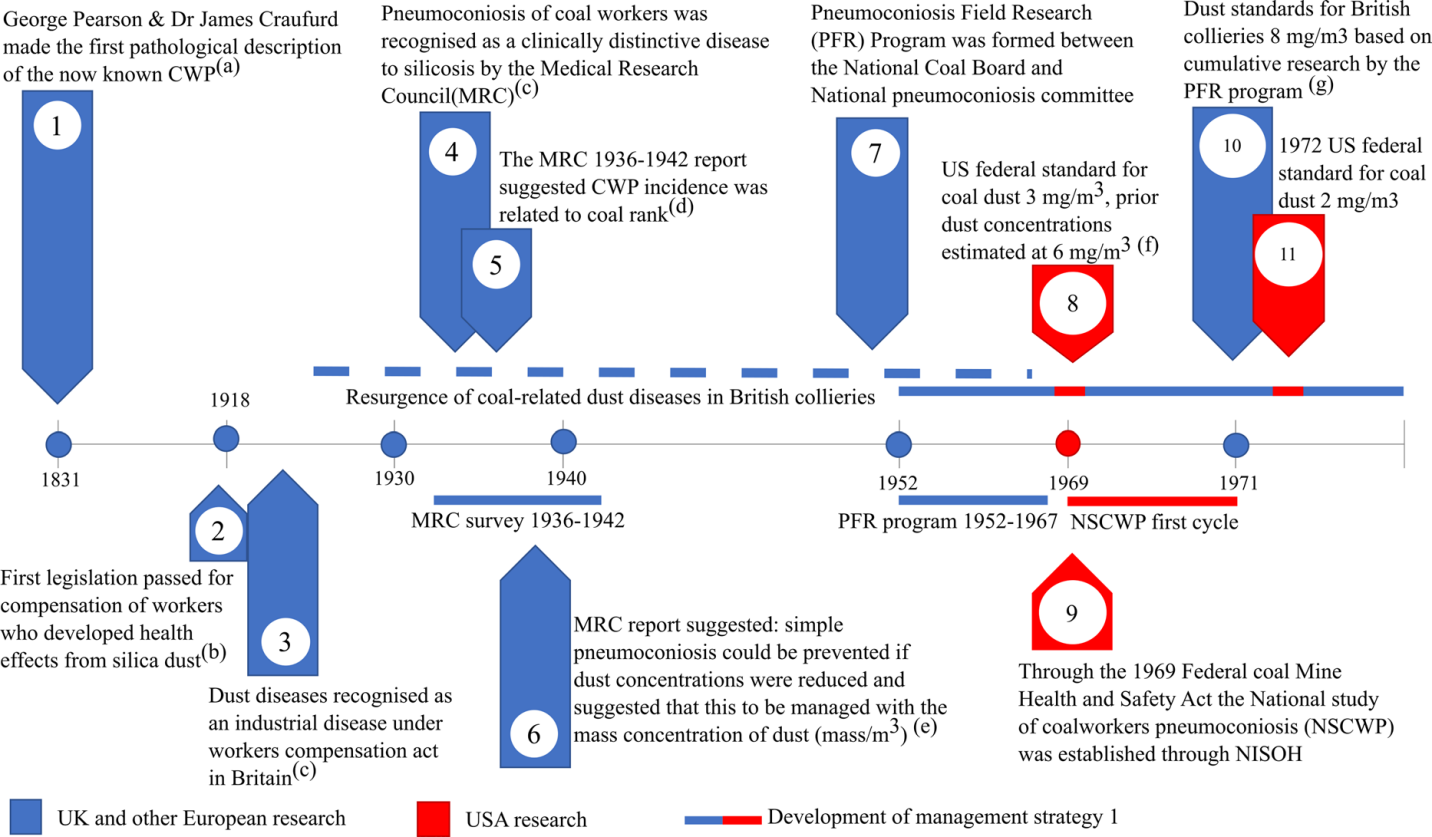
A timeline from 1831 to 1971 that covers the historical development of strategies for coal mine dust diseases. Primary references for the points mentioned are as follows: (a) Donaldson et al. (2017), (b) Bufton and Melling (2005), (c) Heppleston (1992), (d) D’Arcy Hart and Tansey (1998), (e) D’arcy Hart and Aslett (1942), (f) Attfield and Morring (1992a), and (g) Jacobsen et al. (1970)

Research on the management of coal mine dust-related diseases has spanned over 60 years, involving stakeholders from various sectors. These include the government, industry parties, and research institutes—predominantly from both Europe and the USA. Some of the major events from this timeline include the first pathological description of what we now know as coal workers’ pneumoconiosis (CWP) in 1831 (Donaldson et al., 2017) (Fig. 1 timepoint 1). For a considerable period between 1831 and the mid-1900s, CWP was thought to be a form of silicosis. This led to what Heppleston (1992) (Fig. 1 timepoint 3) referred to as “decades of doubt”, where it was unclear as to whether the coal mine dust disease observed in 1831 could be considered a clinically distinctive disease.

However, between 1936 and 1942 the Medical Research Council (MRC) in Britain conducted an epidemiological survey to understand the nature of the resurgence in coal mine dust disease among workers (Fig. 1 timepoints 4–6). During this period, the disease was termed pneumoconiosis of coal workers, a clinically distinctive disease to silicosis (D’Arcy Hart & Tansey, 1998). As part of the research efforts, a report was produced by Hart and Aslett (1942) which formed the basis for the first strategy for reducing the cases of CWP (Fig. 1 timepoint 6). They suggested that CWP would not progress if dust concentrations were under a certain level. Furthermore, it was proposed that a limit could be set based on a value of the mass concentration of dust in the air (mg of dust/m^3^ of air). Apart from commenting on the management of CWP, it was suggested that the differing prevalence levels between mines could be related to the rank of the coal.

Following the results of the MRC survey, a second epidemiological survey termed the Pneumoconiosis Field Research (PFR) programme was initialised in 1952. During this period, research was conducted to define safe dust limits for British collieries. Concurrent with this, the USA implemented legislation to curb the burden of CWP cases at American collieries. This resulted in the implementation of dust limits and the initiation of the national epidemiological survey to understand the state of CWP in the USA (Fig. 1 timepoints 8, 9).

**Fig. 2:**
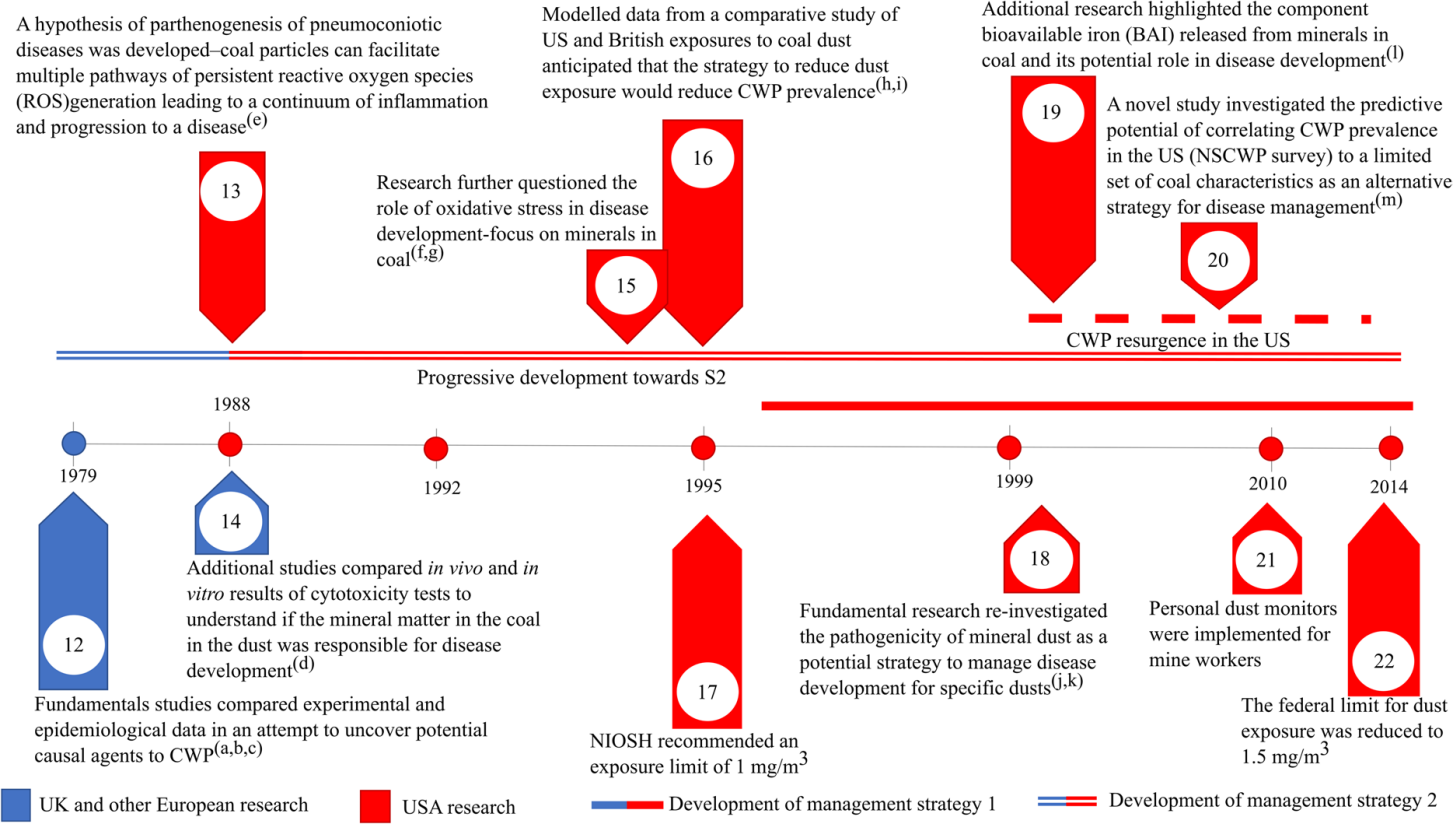
Timeline from 1979 to 2014 continuing the historical development of strategies for coal mine dust diseases from Fig. 1. Primary references for points mentioned are as follows: (a) Gormley et al. (1979), (b) Bennett et al. (1979), (c) Reisner et al. (1982), (d) Le Bouffant et al. (1988), (e) Vallyathan et al. (1988a), (f) Vallyathan (1994), (g) Huang et al. (1994), (h) Attfield and Morring (1992b), (i) Attfield and Seixas (1995), (j) Vallyathan et al. (1998), (k) Huang et al. (1999, 2002), and (m) Huang et al. (2005)

As part of the research collated by the PFR programme, a dust limit of 8 mg/m^3^ was suggested for collieries in 1970 based on British mines (Jacobsen et al., 1970) (Fig. 1 timepoint 10). Upon implementation of the standards, which resulted in a dramatic reduction of dust, Britain further opted to remove miners with detected issues to “less risky” areas of the mine and limit the time of work shifts (D’Arcy Hart & Tansey, 1998). By contrast, the USA prioritised the refinement of dust limits which were further lowered to 2 mg/m^3^ in 1972 (Fig. 1 timepoint 11). For the period directly after the intervention, both approaches reduced the incidence of CWP over time and were widely implemented across the globe (Blackley et al., 2018). However, several European countries reported regional discrepancies in the prevalence of CWP despite comparable dust concentrations (Davis et al., 1982). Stemming from the unexplained discrepancies, several studies investigated potential links between epidemiological and experimental results to understand if a causal component in the coal could be identified (Bennett et al., 1979; Le Bouffant et al., 1988; Gormley et al. 1979; Reisner et al., 1982) (Fig. 2 timepoint 12).

In 1988, a hypothesis for the pathogenesis of CWP was proposed stating that coal particles have the potential to persistently generate reactive compounds which leads to a continuum of inflammatory responses and subsequent disease progression (Vallyathan et al., 1988a) (Fig. 2 timepoint 13). This hypothesis elicited several studies postulating the role of minerals in generating reactive compounds and subsequently led to the idea that certain dust may possess inherent toxicity based on their mineralogical and chemical properties (Huang et al., 1994, 1999, 2002; Vallyathan, 1994; Vallyathan et al., 1998) (Fig. 2 timepoints 13, 15, 18, 19). A study in 2005 tested the application of predicting CWP prevalence with chemical compounds, namely bioavailable iron released from coals (Huang et al., 2005) (Fig. 2 timepoint 20). This provided what can be considered the first demonstration of an alternative strategy to identify the pneumoconiotic potency of coal prior to mining.

**Fig. 3:**
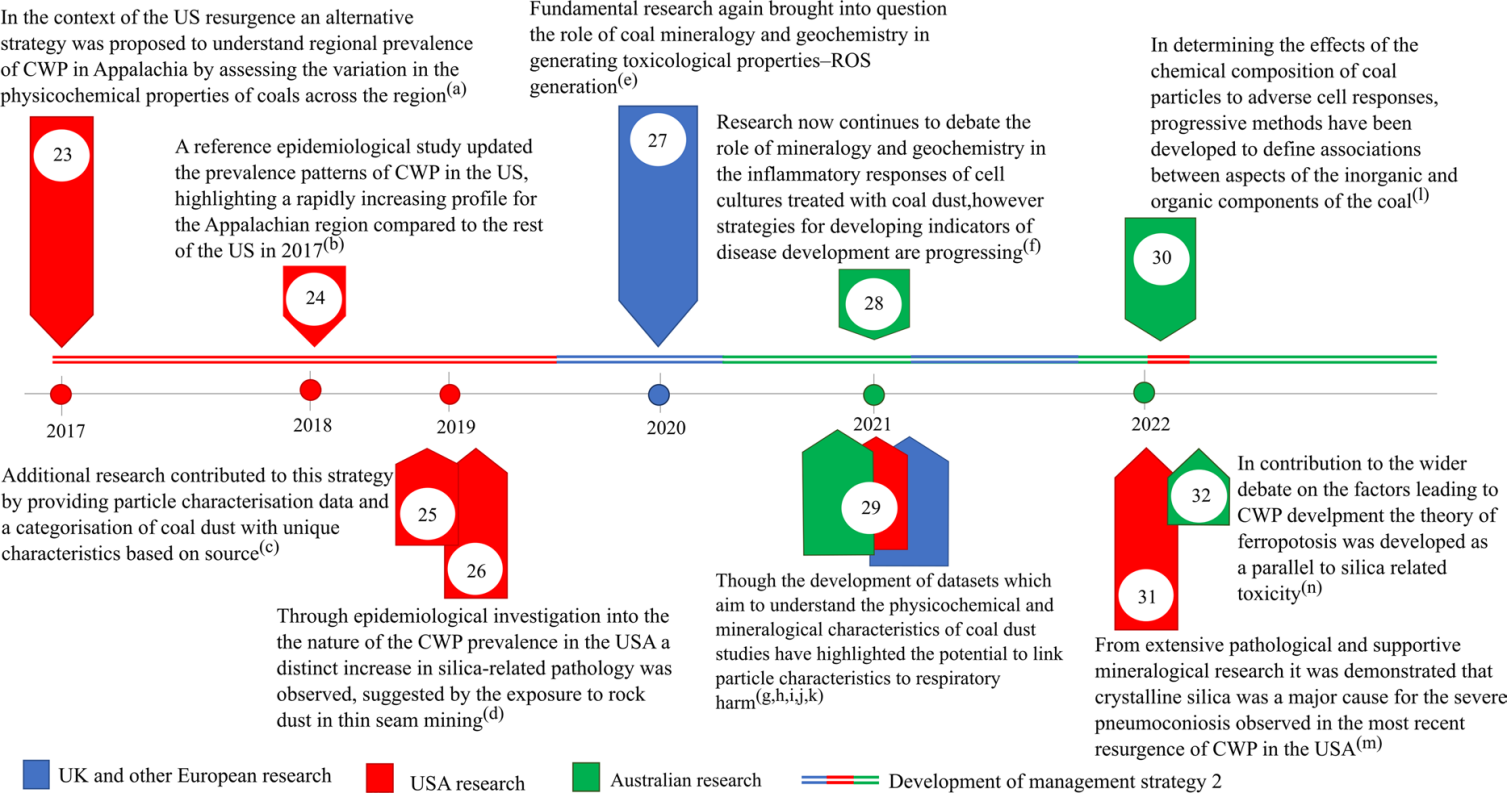
Timeline from 2017 to 2022 continuing the historical development of strategies for coal mine dust diseases from Figs. 1 and 2. Primary references for points mentioned are as follows: (a) Johann-Essex et al. (2017), (b) Blackley et al. (2018), (c) Sarver et al. (2019), (d) Hall et al. (2019), (e) Trechera et al. (2020), (f) Zosky et al. (2021), (g) Trecheraet al. (2021a), (h) Trechera et al. (2021b), (i) Pan et al. (2021), (j) Sarver 2021, (k) Song et al. (2022), (l) LaBranche et al. (2021), (m) Cohen et al. (2022), and (n) Sun et al. (2022)

Despite dust concentration limits at collieries in the USA being reduced to 1.5 mg/m^3^ in 2014 (Fig. 2 timepoint 22), the prevalence of CWP was on an increasing trend, particularly in Appalachia (Blackley et al., 2018) (Fig. 3 timepoint 24). This led to research focused on understanding the physicochemical characteristics of the coal mine dust to shed light on the aspects of the dust which could potentially be linked to CWP and other health effects (Johann-Essex et al., 2017; Sarver et al., 2019) (Fig. 3 timepoints 23, 25). From this work, it was established that the mineralogical and physical characteristics of coal mine dust could vary both between geographical locality and between point sources derived from primary processing streams (Johann-Essex et al., 2017; LaBranche et al., 2021; Pan et al., 2021; Sarver et al., 2021) (Fig. 3 timepoints 23, 29), suggesting that not all coal mine dust can be considered equivalent in terms of its characteristics. Additionally, studies suggested that exposure to silicate minerals derived from cutting into the wall rock may have an impact on the prevalence of coal mine dust diseases, based on an increase in silica-related pathologies in Appalachia (Cohen et al., 2022; Hall et al., 2019) (Fig. 3 timepoints 31, 26). Later studies by Keles et al. (2022) and Keles and Sarver (2022) provided additional characterisation data on the abundance and sources of quartz in coal mine dust within USA collieries. Their results demonstrated that coal mine dust resulting from activities which cut into the rock strata (such as roof bolting) produced elevated levels of quartz compared to the dust produced from run of mine coal. Furthermore, their results showed that, on a regional scale, mines which practice thin seam-mining tended to display elevated levels of quartz in the sampled coal mine dust.

### Modern perspectives on the mitigation of coal mine dust diseases

Following the progression of the resurgence of CWP in the USA, research addressing the health effects of coal mine dust shifted to elucidate the factors relating to the development of coal mine dust diseases (Harrington et al., 2013; Huang & Finkelman, 2008; Shangguan et al., 2022; Song et al., 2022; Sun et al., 2021). By investigating the resulting particle–cell effects, a strong focus has been placed on relating the physicochemical and mineralogical characteristics of coal mine dust to their potential for generating immune/toxic responses. As coal is a chemically complex material, several characteristics related to its composition have been shown to induce tissue damage and serve as potential predictors for CWP risk. In particular, the established mechanism for toxicity and inflammation induced from quartz and its prevalence in coal has commonly been drawn upon when discussing predictors for the harmfulness of coal mine dust (Castranova, 2000; Fubini, 1998). Studies such as Cohn et al. (2006a), Harrington et al. (2012), and Schoonen et al. (2010) have also demonstrated the role of pyrite and bioavailable iron as additional agents responsible for tissue damage via mechanisms independent to quartz. As a result, both pyrite and bioavailable iron have been debated as potential predictors for CWP development. However, the prevalence of silicosis-type CWP, observed by miners post-resurgence in the USA, has brought into question the relevance of iron-bearing minerals over quartz in CWP development (Cohen et al., 2022; Hall et al., 2019) (Fig. 3 timepoints 31, 26).

Studies have compared the bulk mineralogy and geochemistry of sampled coal mine dust to chemical markers of toxicity to elucidate and demonstrate the mechanistic effects of coal mine dust composition on toxicity (Trechera et al., 2020, 2021a, 2021b) (Fig. 3 timepoints 27, 29). Through such analyses, multiple sets of mineralogical and geochemical components were found to contribute to the expression of toxic responses. However, not all the components identified can be attributed to mechanistic pathways relevant to CWP development. Correlations defined by Song et al. (2022), Trechera et al. (2021b), and Zazouli et al. (2021) have demonstrated, in particular, that the pyrite and iron content in coal mine dust can be considered a strong determinant for oxidative potential (an indicator of cellular oxidative stress). Building on this body of work suggesting the relevance of pyrite and bioavailable iron as a significant predictor for CWP development, a pathogenic pathway termed “ferroptosis” was linked to the development of CWP (Sun et al., 2022) (Fig. 3 timepoint 32). In defining this pathway, in vitro tests confirmed the relevance of iron derived from pyrite, as well as additional iron-bearing minerals, as a strong explanatory factor for both cytotoxic and pro-inflammatory responses in lung cells, in the presence of quartz. Despite these recent advancements, there is still no clear understanding or means to holistically compare the relative impact of the various characteristics on the toxic/inflammatory responses observed in mechanistic studies. Consequently, many studies consider the individual strength of characteristics as a potential predictor, but do not address the potential for mechanisms to co-occur or progress at varying magnitudes based on the properties of the dust.

This review aims to provide a means of contextualising the debate on the potential causal factors relating to CWP development by synthesising and integrating the current understanding of coal mine dust as an inherently harmful material and defining the generalised pathways leading to cellular stress and damage post-exposure. Through establishing these concepts, the review further aims to critically assess the physicochemical characteristics of coal mine dust which should be considered as predictors for the development of CWP and other coal mine dust-related diseases based on an integrated understanding of particle toxicology and the geo-anthropogenic nature of the dust. Lastly, the review provides a synthesis of the approaches developed to define the pneumoconiotic potency of coal mine dust and further demonstrates the potential usefulness of coal mine dust characteristics as a tool to assess respiratory harm.

## Coal as a geo-anthropogenic dust source

In the mining environment, unwanted dust is generated as a by-product of several anthropogenic operations. To understand the overall dust burden within collieries, several studies have applied geospatial methods such as satellite imagery to define the amount of dust generated from these sources (Ghose & Majee, 2000; Huertas et al., 2014; Mandal et al., 2012). Collectively, the results from these studies highlighted operations such as ore excavation, transportation, and primary processing of the material as major contributors to the burden of dust within and around collieries. As each of these operations has the potential to impact the inherent characteristics of the dust and thus their potential toxicity, studies have investigated the variability within intra- and/or inter-colliery settings for their potential to produce characteristically distinct particle populations (Johann-Essex et al., 2017; LaBranche et al., 2021; Sarver et al., 2021; Trechera et al., 2021b).

In the context of underground mines, studies by Johann-Essex et al. (2017), Sarver et al. (2021), and LaBranche et al. (2021) have shown that the mineralogical composition of the coal mine dust can vary significantly based on the mine location and operation. Between the different mines investigated, these studies showed that the coal mine dust from cutting sites contained higher proportions of minerals relative to carbonaceous matter. Consistent with the previous observations of Johann-Essex et al. (2017), Sarver et al. (2021), and LaBranche et al. (2021), a study by Trechera et al. (2020) found that the proportion of mineral matter relative to carbonaceous matter varied between sampled coal mine dust. Moreover, some of the dust samples displayed compositional abundances similar to the parental coal, whereas other sampled dust was enriched in mineral content compared to their parental coal. To account for this variation, these studies cited both geogenic variability (such as differences in the regional geology) and anthropogenic activities such as rock dusting and cutting into wall rock. Apart from composition, the results presented by Johann-Essex et al. (2017) and LaBranche et al. (2021) showed that the particle size distributions across different point sources varied both within and between the underground mines investigated. Specifically, the dust collected near cutting zones and return airways (extractive ventilation near cutting) tended to yield higher proportions of finer particles than sites near the intake airways. Apart from coal and mineral dust, both studies in addition to studies by Pan et al. (2021) and Sarver et al. (2021) noted the presence of diesel exhaust particles in the submicron fraction of some mines. Such results highlight the heterogeneous nature of coal mine dust. In assessing whether different cutting methods have an impact on particle size, a study by LaBranche et al. (2021) found that the locality of the mine from where the dust was sampled had a stronger impact on the size distributions than the cutting method (longwall versus continuous miner). In expanding on reasons for this unexpected result, the study proposed that potential differences in geology and the mining conditions (cutting speed, deterioration of cutting pick and dust suppression) may influence the particle size distributions resulting from the different cutting methods.

To assess the impact of various mining operations in an open cast environment, the intra-colliery variability between the characteristics of coal mine dust sourced from several locations in a pit was compared (Trechera et al., 2021b). The results showed that each operation or activity produced distinctly different distributions of particle size and chemical compositions, similarly to the results obtained from the underground mines. Specifically, dust produced from tailings handling and by truck traffic tended to consist of more minerals than carbonaceous matter. This was compared to the dust produced at the coal work fronts, which was predominantly carbonaceous matter. Across the different sites, the particle size of the dust sampled at the coal work front was found to be coarser compared to the dust produced from tailings handling and by truck traffic. To explain this, studies by Amato et al. (2010) and Colinet et al. (2021) proposed that the frequent spraying of dust at the work front could have promoted agglomeration. This contrasts with the tailings handling zone which is not sprayed and is thus not affected by additional moisture.

Ultimately, these findings indicate that the physicochemical and mineralogical properties of coal mine dust are a function of both the anthropogenic activities on collieries which produce the particles and the geogenic properties of the parental coal. Apart from coal mine dust derived from extractive operations, little is known about the anthropogenic dust generated from the stored wastes of the coal beneficiation process. As coal beneficiation waste products are generally enriched in mineral matter (clays, quartz, and sulphides) and subsequently deposited in tailings zones (Oliveira et al., 2012a, 2012b; Tambwe et al., 2020), understanding and managing dust derived from these zones may be important to mitigate the health effects seen by individuals proximal to mines.

## The relationship between coal mine dust, cellular stress, and inflammation

### The fate of inhaled coal particles

**Fig. 4:**
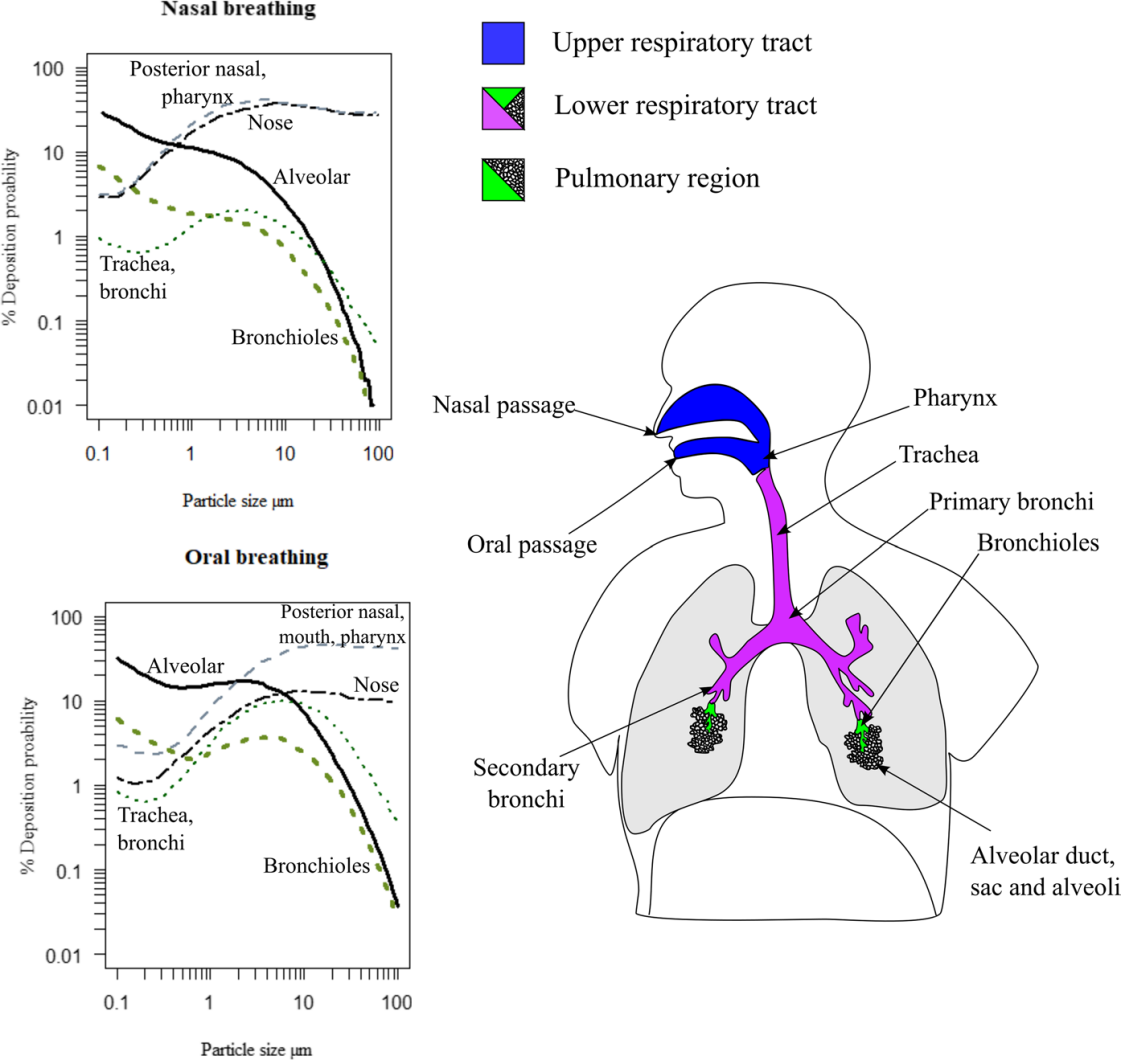
Deposition profiles particles of particles sized between 0.1 and 100 µm under conditions of normal nasal breathing and oral breathing after light exercise, graphs adapted from Bair (2000)

Once a particle has been inhaled, its ultimate deposition in the respiratory tract is understood to be based on the individual’s breathing pattern (through either the nose or mouth) as well as the aerodynamic diameter of the particles (McClellan, 2000; Schulz et al., 2000; Yeh et al., 1976) (see Fig. 4). In addition to these factors, further studies have outlined the role of geometric size, shape, and morphology in the sedimentation and entrainment of particles in lung tissue (Fubini & Fenoglio, 2007; Hassan & Lau, 2009; Muhle & Mangelsdorf, 2003; Plumlee et al., 2006). To further assess the translocation and potential dose of inhaled particles, the International Commission on Radiological Protection Human Respiratory Tract Model was developed (Bair, 1991, 2000). As part of the outputs of this dosimetric model, the probability of deposition for particles of a given size can be interpreted within various regions along the respiratory tract (as demonstrated in Fig. 4). To account for differences in breathing patterns, normal nasal breathing and oral breathing after light exercise were examined as conditions to contextualise the deposition environment. While the model was developed for radionuclide particles, it has been widely cited as a tool to assess the deposition profiles of airborne particles along the respiratory tract (Haddrell et al., 2015; Kodros et al., 2018; Maynard & Kuempel, 2005; Oberdörster, 2000; Patel et al., 2020; Plumlee et al., 2006).

Depending on where the particles are deposited, a variety of mechanisms may be employed as a part of the body’s protective strategies against foreign material (Lauweryns & Baert, 1977; Plumlee et al., 2006; Wanner et al., 1996). In the upper and lower respiratory tract (up to the bronchioles), particles are mostly cleared mechanically via the mucociliary escalator. By trapping the inhaled particles in a mucus coat, particles are then moved upward by ciliated epithelium where they are eventually swallowed. This process prevents most particles from encountering the sensitive alveolar region and potentially entering the bloodstream through the air-blood boundary. However, several studies have established that fine (< 10 µm) and ultrafine (< 1 µm) particles—particularly particles which are poorly soluble in lung fluids—have the potential to penetrate deeper into the lung, particularly the alveolar region (Donaldson et al., 2002; Kroll et al., 2011; Muhle et al., 1990; Nel et al., 2006; Oberdörster, 2000; Oberdörster et al., 1994). In the context of coal mine dust, studies such as Gonzalez et al. (2022a), LaBranche et al. (2021), Pan et al. (2021), and Sarver et al. (2021) have generally found that respirable particulates range in size from 5 to 1 µm. In parallel with these findings, studies by Shangguan et al. (2022) and Trechera et al. (2021a, 2021b) measured the distribution of particle sizes from deposited coal mine dust to obtain the volume percentage of particles in different size classes. Collectively, their results showed that 10–30% of deposited dust collected is < 10 µm, while 2–25% of the dust reports < 4 µm. As the content of coal mine dust is poorly soluble but potentially biologically reactive, such proportions of particles in these size classes highlights the necessity to understand how different physicochemical properties impact cellular activity and the general pulmonary environment.

**Fig. 5:**
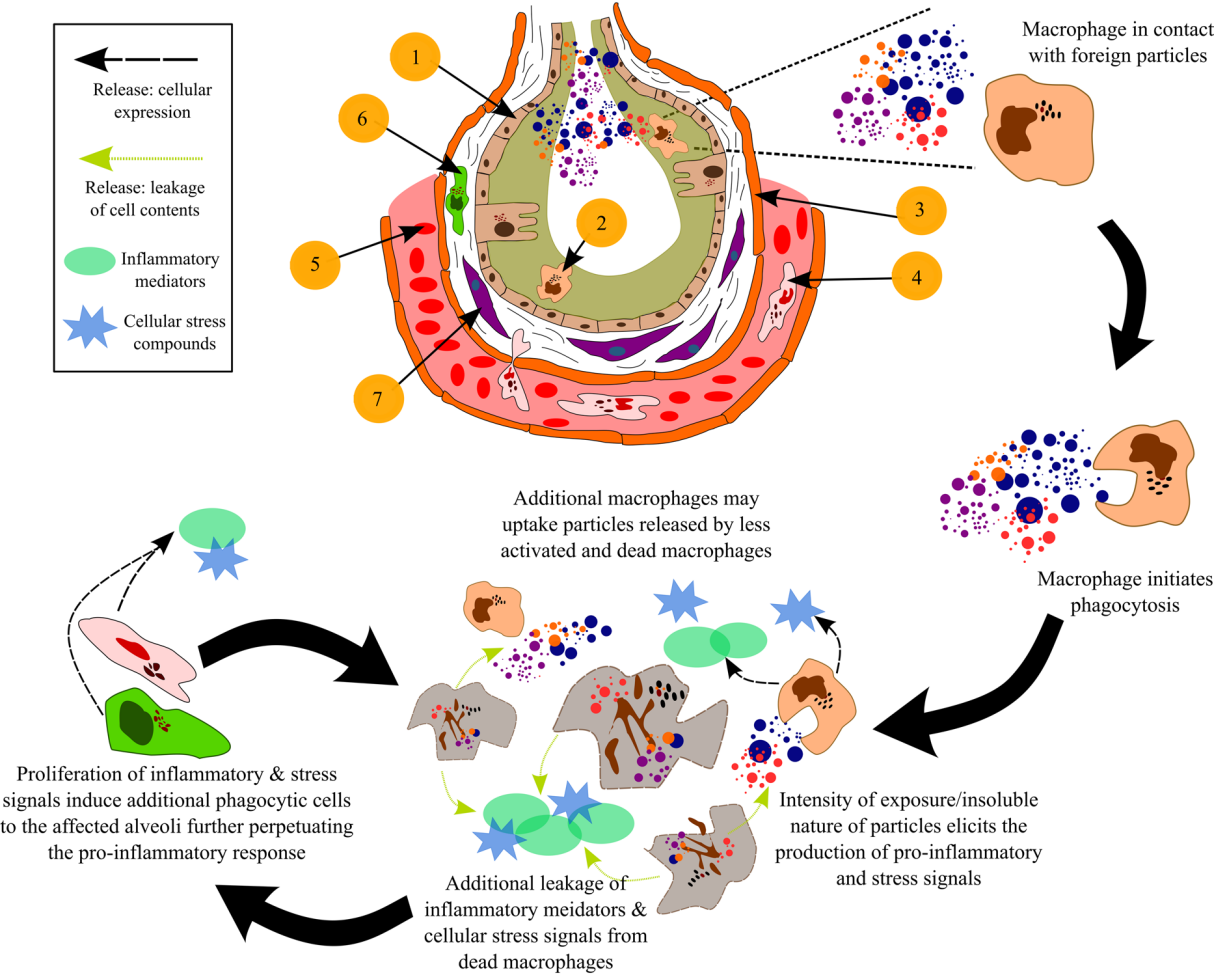
Structure of the alveoli and progressive cycle of inflammation derived from macrophages. Description of cells and tissue present (1) alveolar epithelial cells lined by a surfactant—olive green. (2) Alveolar macrophage (phagocytic cell). (3) Endothelium—orange, and capillary—pink space containing red blood cells. (4) Neutrophil (type of white blood cell) present in an adjacent capillary. (5) Red blood cells present in the pulmonary capillary. (6) Additional macrophage cells present in the interstitium (collection of support tissue including the epithelial cells). (7) Fibroblasts (cells that synthesise collagen and produce connective tissue) present in the interstitium

In the instance where foreign particles have made their way to the alveolar region, phagocytic cells (e.g. macrophages) are activated to remove the contaminant. At a high exposure, the phagocytes can become overwhelmed and secrete signals to mobilise additional cells of immune system by inducing further pro-inflammatory responses (see Fig. 5). In addition to deposition, the composition of the particles has also been found to impact the biogeochemistry of the alveoli, triggering the macrophages and epithelial cells to release stress signals and pro-inflammatory mediators (Borda & Schoonen, 2001; Harrington et al., 2013; Leung et al. 2012; Orona et al., 2014; Sun et al., 2022). These mechanisms emphasise the need to develop an understanding of the cytotoxic and immunological implications of particle–cell interactions based on the inherent properties of inhalable particles to address this knowledge gap.

### The role of oxidative stress and inflammation in the pathogenicity of coal mine dust

**Fig. 6:**
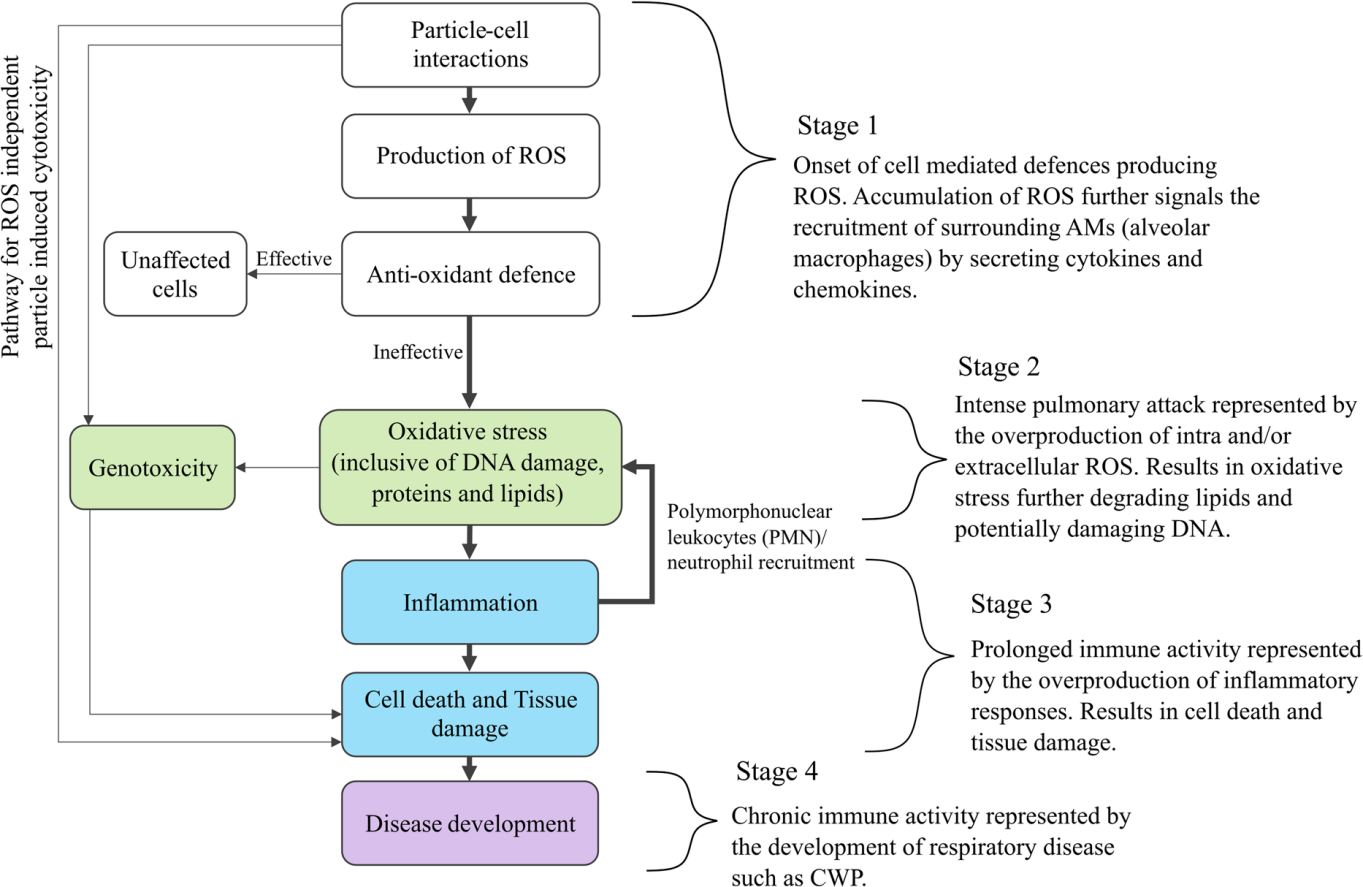
A generalised depiction of the disease induction pathways from particle exposure based on Roesslein et al. (2013)

In outlining the pathogenicity of coal mine dust, markers of pulmonary damage have been characterised to follow a set of stages leading to the development of diseases such as CWP (Castranova, 2000; Schins & Borm, 1999; Vallyathan et al., 1998)—outlined in Fig. 6. Starting from the initial deposition, particles settle into alveolar fluid secreted by epithelium cells (see Fig. 4). Once in the fluid, the nature and chemistry of the particle surface have the potential to interact with compounds such as molecular oxygen, releasing highly reactive chemicals known as reactive oxygen species (ROS)—Stage 1 in Fig. 6. The presence of both the particles and these reactive compounds activates the alveolar macrophages, which travel to the sites of deposition to ingest the particles via phagocytosis. In cases where the number of particles or chemical reactivity of the material has not overwhelmed the system, the abundance of ROS is counteracted by antioxidant compounds secreted by macrophages. These compounds ultimately mediate the potential for ROS to damage healthy cells and tissue. However, when the exposure is acute, severe damage may occur to both the macrophages and epithelial cells via direct and indirect means (Michael et al., 2013; Orona et al., 2014; Yang et al., 2009). As a result, pathways leading to the direct cytotoxicity and indirect damage of DNA (genotoxicity) can be enacted (Könczöl et al., 2011; León-Mejía et al., 2016; Roesslein et al., 2013).

Although both direct and indirect cellular damage is understood to play a role in the physiological degradation of the lung, studies have shown that the overproduction of ROS and subsequent development of oxidative stress is most likely the dominant pathway involved in the development of pneumoconiosis and other coal mine dust-related diseases (Castranova & Vallyathan, 2000; Maanen et al., 1999; Pinho et al., 2004; Schins & Borm, 1999; Vallyathan et al., 1998; Zhang & Huang, 2002; Zhang et al., 2002)—Stage 2 in Fig. 6. In addition to ROS generated from alveolar cells, it has been demonstrated that redox reactions between transition metals (at the surface of minerals and from metal leaching) and minerals such as quartz can produce highly reactive hydroxyl radicals (Cohn et al., 2006a, 2006b; Dalal et al., 1990, 1995; Ghio & Quigley, 1994; Schoonen et al., 2010; Vallyathan et al., 1988a, 1998; Winterbourn, 1995). In this context, the cause to tissue damage and eventual fibrosis is attributed to cycles of non-specific damage of cell membrane phospholipids via lipid peroxidation, consequently leading to the release of inflammatory mediators known as cytokines and chemokines and the recruitment of additional inflammatory cells (polymorphonuclear leucocytes (PMNs)/ neutrophils) (Borish & Steinke, 2003; Hiraiwa & Van Eeden, 2013; Laskin, 2009; Rice-Evans, 1994; Vanka et al., 2022)—Stage 3 in Fig. 6.

## Physical particle characteristics relevant to cellular stress and inflammation

### Size

In addressing the importance of particle size relative to inflammation and lung injury, studies by Morrow (1988) and Muhle et al. (1990) hypothesised that the volumetric loading of highly insoluble ultrafine particles could impair the ability of macrophages to further internalise particles and thus hinder the removal of particles in the alveoli. Under such circumstances, Morrow (1988) proposed that this occurs when approximately 6% of the normal macrophage volume is filled by phagocytised particles. As a result of impaired clearance, the build-up of particles may occur and can subsequently lead to direct and indirect damage to macrophage and epithelial cells depending on the chemical reactivity of the particles (Broug-Holub et al., 1997; Donaldson et al., 2000; Lison et al., 1997; Warheit et al., 1997). Furthermore, the particles may translocate into the bloodstream where they can potentially impact the cardiovascular system (Decuzzi et al., 2010; Wallenborn et al., 2007).

To assess the dependency of particle sizes on the entrainment of particles within the lung, as well as associated inflammatory damage in vivo, a study by Oberdörster et al. (1994) exposed groups of rats to two size fractions (< 250 nm and < 20 nm) of highly insoluble TiO_2_ particles under equivalent doses. To compare the effects of the different size fractions, a group of rats were only exposed to filtered air. The findings showed that, over the same period, the < 250-nm particles were retained in the alveolar space for twice as long as the control (where the retained particles in the macrophages reached ~ 9% of the macrophage volume). In comparison, the < 20-nm particles were retained four times longer in the alveolar space relative to the control (where the retained particles in the macrophages reached ~ 2.6% of the macrophage volume). Based on these results, Oberdörster et al. (1994) challenged the cut-off phagocyte internalisation volume proposed by Morrow (1988) and further suggested that the size of particles can inhibit the internalisation of particles by macrophages to different degrees irrespective of the phagocytised volume of the cell. In this context, it was presumed that effective macrophage removal would result in the migration of cells to the upper respiratory tract via the mucociliary escalator—further described by Wanner et al. (1996).

By investigating the dependency of particle size on inflammatory responses at a dose congruent with particle overload, the results from Oberdörster et al. (1994) showed that the < 20-nm particles displayed a greater influx of recruited monocytes and polymorphonuclear leucocytes (PMNs) and secreted more proteins (such as lactate dehydrogenase indicating cytotoxicity from cytosolic damage) than the < 250-nm particles of the same dose. These results highlighted that the effective dose in alveoli is not a consistent predictor of impaired clearance or inflammation where there is a distribution in particle sizes. To complement these results, the inflammatory influx of PMNs was compared to the surface area of the retained particles. Through this relationship, both < 20- and < 250-nm particles could be explained by a common dose–response curve. This suggests that the surface area of retained particles may be a more useful determinant for impaired macrophage clearance and inflammation than retained dose. These observations were supported by similar studies on chemically non-reactive particles such as rutile, polystyrene, diesel, and carbon-based soot which showed a common dose–response relationship between the surface area/number of particles and the influx of PMN and other inflammatory indicators (Brown et al., 2001; Lison et al., 1997; Maynard & Kuempel, 2005; Oberdörster et al., 2005; Stoeger et al., 2006; Tran et al., 2000).

Regarding the effect of size of inflammation by more biologically reactive particles, a study by Mischler et al. (2016) investigated the level of mitochondrial ROS and TNF-α (an inflammatory indication) expressed by murine macrophages across different size fractions of quartz. Their results showed that particles in their coarsest class (4 µm) displayed a consistent relationship between mitochondrial ROS generation and TNF-α release from acute exposures (2-, 4-, and 8-h timepoints). However, the results for finest fraction (0.3 µm) showed that the finer particles were able to be internalised by macrophages at a higher rate. This in turn translated to higher rates of mitochondrial ROS production and TNF-α release from the finer particles after 4 h compared to the coarser particles. This could suggest that the activity of biologically reactive particles such as quartz could be exacerbated by their particle size.

### Surface area and reactivity

From the perspective of particle reactivity, observations have shown that an increase in the specific surface area of particles allows for a greater proportion of atoms/molecules to bind and react at the surface (Oberdörster et al., 2005). However, such an outcome could arise either from a reduction in particle size or by a change in the aspect ratio, both of which have been shown to elicit independent pathways to the production of reactive oxygen species (ROS) and inflammatory mediators (O’Neill, 2008; Oberdörster et al., 1994).

In the context of coal mine dust pathology, the surface reactivity of free minerals and coal-mineral composites has been observed to strongly influence the production of toxic hydroxyl radicals (Fubini et al., 1990; Schoonen et al., 2010; Zhang et al., 2018). This is based on in situ reactions between surface functional groups and metal complexation sites enabling transition metal-based redox reactions. However, the contribution of these surface-based reactions to the overall ROS burden remains unclear. In addition to these reactions, defects at the surface of crystalline matter (induced by grinding) have been shown to serve as sites for free radical generation (Schoonen et al., 2006). In the context of mining and mineral processing, such observations may be occupationally relevant as a harmful geo-anthropogenic alteration to dust generated from mechanical crushing. By investigating the effect of grinding on poorly soluble minerals such as quartz, studies have found that these freshly fractured particles not only generate hydroxyl radicals, but also display higher rates of cytotoxicity at equivalent doses compared to aged particles (Fubini et al., 1987; Lison et al., 1997; Vallyathan et al., 1995).

In composite particles, the toxic effects resulting from surface reactivity, morphology, and surface area exposure are known to occur simultaneously. Although studies have extensively outlined the individual toxic effects for each of these characteristics (in monomineralic systems), the degree to which these combined factors impact phagocytosis in composite particles is not yet understood.

### Shape

In the scope of physical characteristics related to particle toxicity, the potential impact of particle shape on toxicity and inflammation has been widely acknowledged through research conducted on fibrous minerals (Fubini & Fenoglio, 2007; Maynard & Kuempel, 2005; Plumlee & Ziegler, 2003). However, particle shape has rarely been examined by immunological studies investigating the pneumoconiotic potency of coal mine dust. Currently, it is generally understood that high aspect ratio particle shapes can disrupt the successful internalisation of particles during phagocytosis (Cannon & Swanson, 1992; Schins & Borm, 1999). In this process, termed “frustrated phagocytosis”, affected macrophages generate ROS and inflammatory indicators in response to the failure to successfully internalise a given particle (O’Neill, 2008). As a result of this response, epithelial and fibroblast cells are triggered to produce secondary pro-inflammatory and fibrogenic mediators which may subsequently induce scarring if perpetuated.

**Fig. 7:**
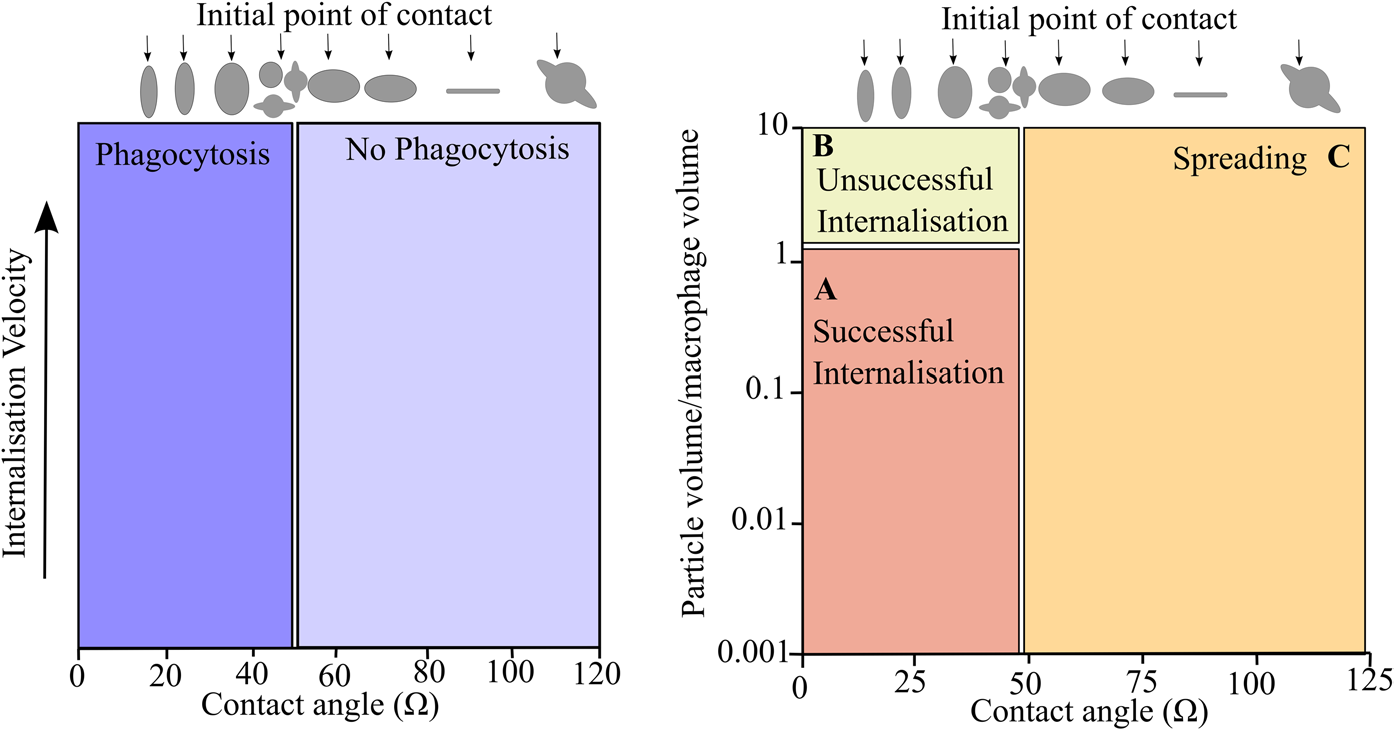
Left-hand side represents the ability of phagocytosis to occur depending on the shape of the particle and the initial contact point. The right-hand side displays a phase diagram highlighting the different responses macrophages display post-initiation of phagocytosis, where area (A) represents a complete engulfment of the particle by the macrophage, area (B) represents the incomplete engulfment, and area (C) represents the spreading of the macrophages over the particle surface rather than engulfment—based on Champion and Mitragotri (2006)

Apart from the research conducted on fibrous particles, a study by Champion and Mitragotri (2006) determined that various non-spherical particle shapes can impact the functioning of macrophages, leading to frustrated phagocytosis. Based on their findings, it was determined that phagocytosis could be initiated for a range of particle shapes with equal size, surface area, and chemistry (see Table 1 for a description of the particle shapes). Their results further indicated that, depending on the initial contact point (flat versus a curved surface), complete phagocytosis may not occur, irrespective of the size of the particle relative to the cell volume. By defining the contact point as the angle between the macrophage membrane and the point of contact on the particle surface, they were able to generalise the relationship between shape and phagocytosis (displayed in Fig. 7). Based on this relationship, the rate of phagocytosis was found to decrease when the angle of the contact point was less than 45^°^. For a contact point with an angle greater than 45^°^ (which represents a flat surface or concave contact point), no change in the rate of phagocytosis was observed. The results further demonstrated that, for particles with equivalent ratios of particle volume to macrophage volume, the angle of initial contact was found to determine whether complete internalisation or macrophage spreading would occur (see Fig. 7). In this context, macrophage spreading was induced where the contact point was initiated at flat surfaces and concave points. In contrast to this observation, complete phagocytosis was found to occur when the macrophages encountered spheres, dome-/ring-shaped particles, and the rounded edges of the ellipsoids.

**Table 1:** Representation of the top and side view of the particle shapes represented in Champion and Mitragotri (2006)

Sphere	Oblate ellipsoid	Prolate ellipsoid	Elliptical disc	Rectangular disc	UFO
Radius: (1.0–12.5 µm)	Major axis: (4 µm) Aspect ratio: (4)	Major axis: (2–6 µm) Aspect ratio: (1.3–3)	Major axis: (2–6 µm) Aspect ratio: (2–4) Thickness: (400–1000 nm)	Major axis: (4–8 µm) Aspect ratio: (1.5–4.5)	Sphere radius: (1.5 µm) Ring radius: (4 µm)

Ultimately, the implications of incomplete phagocytosis amount to the persistence of particles in the lung, as discussed in the section on particle size. As a result of this, biogeochemical reactions between soluble particles and extracellular fluids can continue, further leading to the direct/indirect damage of tissues via radical attack. In this context, particle characteristics which have been demonstrated to impair phagocytic activities (such as particle shape) may serve as strong predictors for pneumoconiotic potency of both poorly and moderately soluble dust. However, in the case of coal mine dust, which has soluble and reactive components, it is unclear what contribution impaired phagocytosis makes to acute inflammatory responses relative to particle–cell biogeochemical reactions, as both processes are likely to occur simultaneously.

## The role of composition and geochemistry in cellular stress and inflammation

### Carbonaceous material

In early epidemiological research on the prevalence of CWP, observations from several mine cohorts found that the prevalence of CWP differed depending on the geographical region (Attfield & Morring, 1992b; Le Bouffant et al., 1988; Hurley et al., 1982; Morgan et al., 1973). In explaining the possible reasons for this discrepancy, the regional differences in coal rank were found to show a limited level of explanatory power in accounting for disease prevalence. Based on this observation, a study by Dalal et al. (1989) attempted to uncover an experimental link between coal rank and CWP. From their results, it was determined that freshly ground coal produces an increasing number of carbon-centred free radicals with increasing rank. By collating these results with the knowledge that free radicals are highly influential in progressing toxic pathways leading to CWP development, studies hypothesised that these carbon-centred radicals could be implicated in oxidative damage and serve as a potential predictor for the differential toxicity of coal mine dust (Dalal et al., 1990; Huang et al., 1999). However, further experimentation showed that these free radicals are highly sensitive to air and displayed low reactivity with oxidising agents, suggesting that these types of free radicals are inert (Huang et al., 1999).

To further assess the role of the carbonaceous matter in the production of hydroxyl radicals from coal mine dust samples, a study by Cohn et al. (2006a) investigated the contribution of hydroxyl radicals produced from mixtures of carbonaceous matter and pyrite (a mineral known to produce ROS through redox chemistry). The results showed that, upon adding consistent quantities of the coal (containing no pyrite) to a fixed quantity of pyrite, no significant change to the levels of hydroxyl radical was detected. This suggested that carbonaceous matter in coals does not play an active role in the generation of highly reactive oxygen radicals. However, studies have also shown that compounds derived from carbonaceous matter can play a supporting role in redox-based reactions by forming a complexation site with transition metals derived from mineral sources (Dalal et al., 1995; Ghio & Quigley, 1994; Ghio et al., 1992). Despite these studies, no definitive conclusion has been reached on the role of the carbonaceous matter in coal and its implication for disease development, with experimental studies to date indicating that it may not play a causative role in the definition of pneumoconiotic potency of coal mine dust.

### Bioavailable iron

Since the discovery of iron concentrated in the lungs of miners with CWP, studies postulated the role of bioavailable iron in the pathology of coal mine dust (Dalal et al., 1995; Ghio & Quigley, 1994). In the context of biogeochemical reactions in the body, transition metals and iron especially have been shown to react with extracellular hydrogen peroxide producing highly reactive hydroxyl radicals via Fenton chemistry (Eq. 1) (Meneghini, 1997; Winterbourn, 1995). While this pathway may seem direct and further limited by the availability of ferrous iron, studies have shown that a high concentration of reducing agents, such as ascorbate, pyruvate, and glutathione, has the potential to facilitate the reduction of ferric to ferrous iron by cycling the ferrous iron and perpetuating the Fenton mechanism (Eq. 2) (Cross et al., 1994; Pritchard et al., 1996; Slade et al., 1985; Sun et al., 2022). Additionally, studies have shown that ferrous iron can facilitate a feedback production of hydrogen peroxide in the presence of oxygen via the Haber–Weiss reaction, which further drives the Fenton mechanism (Eqs. 3 and 4) (Ghio & Quigley, 1994; Kehrer, 2000; Schoonen et al., 2010; Winterbourn, 1995).

1 $${\text{Fe}}_{{({\text{aq}})}}^{2 + } + {\text{H}}_{2} {\text{O}}_{2} \to^{ \cdot } {\text{OH}} + {\text{OH}}^{ - } + {\text{Fe}}_{{\text{(aq)}}}^{3 + }$$

2 $$Fe_{{({\text{aq}})}}^{3 + } + {\text{Reductant}} \to {\text{Fe}}_{{({\text{aq}})}}^{2 + } + {\text{Reductant}}^{ + }$$

3 $${\text{Fe}}_{{({\text{aq}})}}^{2 + } + {\text{O}}_{2} \to {\text{Fe}}_{{({\text{aq}})}}^{3 + } + ({\text{O}}_{2}^{ \cdot } )^{ - }$$

4 $${\text{Fe}}_{{({\text{aq}})}}^{2 + } + ({\text{O}}_{2}^{ \cdot } )^{ - } + 2{\text{H}}^{ + } \to {\text{Fe}}_{{({\text{aq}})}}^{3 + } + {\text{H}}_{2} {\text{O}}_{2}$$

To date, research defining the mechanics of iron-mediated oxidative damage has predominantly focused on the complexation of aqueous iron as the main mode of iron availability (Cohn et al., 2006a; Dalal et al., 1995; Harrington et al., 2012, 2015; Vallyathan, 1994). Regarding the possible pathways for iron complexation, studies hypothesised that “humic-like substances” (HLS) derived from carbonaceous matter could form organometallic complexes with iron and serve as sites to catalyse the generation of ROS (Ghio & Quigley, 1994; Ghio et al., 1992; Pritchard et al., 1996). Under these conditions, it was proposed that continuous cycles of the Fenton mechanism could occur through the reduction of iron by natural reducing agents, perpetuating oxidative damage to lung tissues. Despite these results, the use of HLS as a potential predictor for iron-mediated oxidative damage has not been utilised in the context of coal mine dust toxicity. As studies aim to develop robust particle characterisation datasets of coal mine dust for the interpretation of health outcomes, the quantification of HLS may be a useful proxy for iron/transition metal-mediated damage.

### Reactive mineralogy in coal and its implications for CWP

#### Quartz

The highly toxic nature of inhalable quartz particles has been well established in the literature (Borm, 2002; Fubini, 1998; Fubini et al., 1990; Jelic et al., 2017; Turci et al., 2016). While the molecular mechanisms relating to its toxicity remain unclear, it is accepted that the silanol functional groups, formed on the surface of hydrated quartz particles, can readily produce hydroxyl radicals (Castranova & Vallyathan, 2000). Based on the unique properties of silanol groups and their propensity to produce hydroxyl radicals, quartz was thought to be the primary propagator of radical-related cytotoxicity and inflammation (Castranova, 2000; Fubini et al., 1990).

Although the presence of quartz in coal mine dust has been considered a causal agent in characteristic ROS-dominated pathways leading to CWP, workers exposed to coal mine dust with little to no quartz were still found to develop CWP (Collis & Gilchrist, 1928; Finkelman et al., 2002; Heppleston, 1992; Reisner et al., 1982). Mechanistically, studies found no consistent relationship between quartz content and toxicity when investigating the pulmonary potency of coal-quartz mixtures in vitro and in vivo (Davis et al., 1982; Ross et al., 1962). To account for such results, a study by Le Bouffant et al. (1988) suggested that the presence of clay surface coatings on the quartz was a reason for the lack of a clear relationship between toxicity and quartz in coal mine dust. Subsequent experimentation confirmed this phenomenon and further suggested that quartz particles may exhibit surface aluminosilicate contamination in dusty work environments (Harrison et al., 1997; Wallace et al., 1994). In continuation of the findings by Harrison et al. (1997) and Wallace et al. (1994), studies by Keles and Sarver (2022) and Gonzalez et al. (2022b) have observed the surface coating of quartz in coal mine dust with aluminosilicate clays using SEM–EDS (scanning electron microscopy and electron dispersive X-ray spectroscopy). In this study, they further categorise the different textural associations between quartz and the clays as: clay-occluded quartz, micro-agglomerates containing quartz, and particles embedded with quartz grains. Such results demonstrate the complex textural relationships between minerals in coal mine dust and their potential to impact the level of mineral-related reactivity within the body.

While it is understood that the chemical reactivity of quartz may be variable, studies have found that freshly fractured surfaces of crystalline quartz particles can produce additional free radicals, further adding to the burden of ROS and oxidant-related damage (Fubini et al., 1987; Vallyathan et al., 1995; Vallyathan et al., 1988b). In the context of collieries which mine thin coal seams, the cutting of quartz-rich wall rock, and the concomitant generation of mine dust with a high abundance of fractured quartz particles, was found to induce the development of silica-related r-type opacities within the lungs of miners (Cohen et al., 2022; Hall et al., 2019). Through an investigation into the mechanisms of fractured quartz toxicity, a study determined that the active fracturing of quartz leads to the disorganisation of surface functional groups (Leinardi et al., 2020; Turci et al., 2016). This was suggested to result in the development of reactive silanol patches and surface radicals.

Ultimately, it can be argued that the reactivity of quartz in composite coal mine dust particles (expressed by the abundance of quartz) may not serve as a consistent predictor of oxidative damage or CWP prevalence. This is in part due to the unresolved variability in surface chemistry and potential surface coating of quartz particles by association with clays or though dusting in the environment. However, parameters which can capture the impact of quartz surface fractures, such as specific surface area and surface reactivity, have been suggested as more representative of quartz-related damage than the abundance of quartz (Leinardi et al., 2020; Pavan et al., 2020).

#### Pyrite

For several years, the abundance of pyrite in coal mine dust has been suggested as a potential predictor for oxidative damage and subsequent CWP development (Huang & Finkelman, 2008; Schins & Borm, 1999; Zhang et al., 2002). Through extensive experimentation, studies have established that hydrated pyrite particles in the presence of molecular oxygen can spontaneously generate hydrogen peroxide (Haber–Weiss reaction Eqs. 3 and 4) and hydroxyl radicals (Fenton mechanism Eq. 1) (Borda & Schoonen, 2001; Cohn et al., 2005; Zhang & Huang, 2002; Zhang et al., 2002). Based on these mechanisms, a study by Huang et al. (2005) attempted to map and predict the prevalence of CWP across the USA through correlations with (1) bioavailable iron (BAI) content calculated from pyrite in coal, (2) pyritic sulphur, (3) total iron, (4) quartz content, and (5) coal rank. The data used included CWP prevalence rates from the national epidemiological survey reported by Morgan et al. (1973) and coal quality data from twenty-four mines across seven states, obtained from the USGS (United States Geological Survey). To determine the quantitative values for BAI, it was assumed that all BAI was derived from the oxidation of pyrite in water (displayed by Eq. 5), although it was acknowledged that additional sources of iron could come from both iron-containing carbonates and silicates. As the available data were limited to pyritic sulphur, sulphate, CaO, and Fe_2_O_3_, an assumption was made that 1 mol pyritic sulphur would equate to 0.5 mol BAI (represented in Eq. 6). Based on the geographical comparison of these characteristics, it was observed that while the coal rank, quartz, and calcite content showed little variation, the pyritic sulphur, total iron, arsenic, and nickel were found to vary from the eastern coal regions to the west. This observed pattern matched the disparities in prevalence records for CWP reported across the regions of the National Epidemiological Survey following 1973 (Attfield & Morring, 1992b).

5 $$2{\text{FeS}}_{2} + 7{\text{O}}_{2} + 2{\text{H}}_{2} {\text{O}} \to 2{\text{FeSO}}_{4} + 2{\text{H}}_{2} {\text{SO}}_{4}$$

6 $${\text{BAI}} = [0.5 \times S_{{{\text{py}}}} ]$$

7 $${\text{Adujusted}}\,{\text{BAI}} = [0.5 \times {\text{S}}_{{{\text{py}}}} + {\text{SO}}_{4} - {\text{CaO}}]$$

By correlating the prevalence of CWP with the physicochemical characteristics of coals across the USA, strong positive relationships were found between BAI and pyritic sulphur, more so than total iron (see Table 2). Similar results were subsequently reported in mechanistic studies, which found that pyritic sulphur in the examined coals showed a positive correlation with the concentration of hydroxyl radicals and the degradation of nucleic acids (Cohn et al., 2006b). This further suggests that pyrite may serve as a good predictor for CWP development across coal regions. In addition to the correlations described, the study by Huang et al. (2005) further assessed the effect of pH on BAI by adjusting the BAI content to account for the buffering capacity of coals (see Eq. 7). It was expected that the pH of the hydrated pyrite system would need to be lower than 4.5 after oxidation of the pyrite surface to stabilise the production of soluble iron sulphate, based on an earlier hypothesis by Huang et al. (1994). In contrast to the lower pH system, the hypothesis further outlined that under systems with a pH higher than 4.5, the pyrite surface will be oxidised forming a non-reactive goethite alteration product. The resulting correlations between the adjusted BAI and CWP prevalence showed that the presence of acid-consuming species lowers the predictability of the BAI mechanism, which can further be supported by the hypothesis of Huang et al. (1994)—refer to Table 2.

**Table 2:** Data on the statistical strength of the correlations between the physicochemical aspects of the coal (data from the USGS) and the CWP prevalence rates obtained from the NSCWP 1970 survey presented in Huang et al. (2005)

Physicochemical aspect	Correlation coefficient (*r*)	95% confidence interval	*P* value
**BAI**	**0.94**	0.66 to 0.999	**0.0015**
***S***_**py**_	**0.91**	0.35 to 0.99	**0.0048**
**Fe**_**T**_	**0.85**	0.20 to 0.97	**0.016**
Coal rank	0.59	0.26 to 0.91	0.16
Quartz	0.28	0.55 to 0.82	0.54
Calcite	− 0.18	− 0.78 to 0.60	0.69
*BAI low pH	**0.90**	0.40 to 0.99	**0.006**
*BAI buffered pH	**0.87**	0.25 to 0.98	**0.01**

*S*_py_, pyritic sulphur; Fe_T_, total iron

^*^Low pH, assuming no neutralisation by calcite, high pH, accounting for acid neutralised by calcite

All significant relationships are indicated in bold (*P* value < 0.05)

While the study by Huang et al. (2005) indicated that BAI derived from pyrite could potentially be used as a consistent predictor of CWP prevalence, it was highlighted that the correlations observed did not necessarily represent causation. Additionally, it should be acknowledged that the context used in this analysis predated the current cases of CWP in this region which have been observed by Cohen et al. (2022) and Hall et al. (2019) to be more related to quartz-related CWP. Despite these considerations, subsequent mechanistic studies further concurred on the toxic nature of pyrite based on the Fenton mechanism, the modality of this reaction was mostly discussed in the context of iron leached from pyrite (Cohn et al., 2006a; Harrington et al., 2012, 2013; Sherekar et al., 2022; Sun et al., 2022). At the same time, it is known that these reactions can occur at the surface of pyrite particles (Borda & Schoonen, 2001; Schoonen et al., 2006; Zhang et al., 2018). Currently, there is a limited understanding of the relative contribution of Fenton reactions at the pyrite surface compared to leached Fe in solution. As pyrite-containing coal particles may be deposited on epithelial cells, it is reasonable to assume that the production of hydroxyl radicals from surface-based Fenton mechanisms may cause direct damage to the cells. As a result, the epithelial cells may secrete pro-inflammatory signals leading to the mobilisation of macrophages. Based on this, it can be argued that research on the different modalities of direct and indirect pyrite-induced damage may be beneficial in determining additional predictors for inflammation and tissue damage. Ultimately, studies conducted to date have shown that pyrite in coal mine dust has the potential to both directly and indirectly produce highly reactive hydroxyl radicals. However, it remains unclear what relative impact Fenton-related damage has on a cellular level in relation to other composition or morphological-based mechanisms of toxicity. By finding ways to elucidate its relative impact, the relevance of pyrite and bioavailable iron as a toxic agent in coal mine dust can be understood more holistically.

#### Miscellaneous minerals

Apart from pyrite, a study by Sun et al. (2021) showed that other iron-bearing minerals such as siderite (an iron carbonate) may also play a role in Fenton-related inflammation and cytotoxicity. While pyrite is often found to be the dominant iron-bearing mineral, its reactivity with water and carbonate minerals can lead to the formation of additional non-sulphide minerals, such as iron-bearing sulphates and iron oxyhydroxides under basic conditions. By understanding the different iron-bearing assemblages present in coals, this may assist in determining whether iron may be present in a biologically active form or not.

In the context of quartz-related toxicity, aluminosilicate clays (such as kaolinite) have been found to play a depressive role in the bio-reactivity of quartz surfaces (Tourmann & Kaufmann, 1994; Wallace et al., 1994). However, pure kaolinite has been shown by Davies (1983), Davies et al. (1984), and Wastiaux and Daniel (1990) to induce cytotoxicity in macrophages, although its mechanisms are currently not completely understood. Considering that clays make a substantial proportion of the mineral content in coal mine dust, apart from quartz and pyrite, the potential cytotoxic and depressive effects of kaolinite should be considered in discussions around the pneumoconiotic potency of coals.

## The utilisation of coal particle characteristics to assess potential respiratory harm

The complex reactivity resulting from particle–cell interactions has been well documented for coal mine dust, particularly in relation to composition-driven reactions in the lung. While there is still uncertainty on the relative impact of composition and particle morphology on oxidative stress and inflammation, studies have sought to develop comprehensive datasets on coal mine dust characteristics to quantify known predictors of cellular damage (Johann-Essex et al., 2017; LaBranche et al., 2021; Moreno et al., 2019; Trechera et al., 2020). Although methods exist for the quantification of physicochemical and mineralogical characteristics of coal, no systematic protocol has been developed for the robust characterisation of the physicochemical and mineralogical characteristics of coal mine dust particles.

Despite the lack of a systematic characterisation protocol, studies have attempted to define relationships between coal mine dust characteristics and the potential for oxidative damage using mineral abundances and element distributions (Shangguan et al., 2022; Trechera et al., 2021a, 2021b). Across all these studies, the authors constructed multilinear models to assess relationships between oxidative potential and the two composition-based datasets. While their modelled relationships were found to be consistent with earlier studies that showed the ROS generation potential of pyrite, sulphates, elemental iron, and, to a lesser extent, quartz, the issue of collinearity between characteristics was not addressed. As coal mine dust may contain primary and secondary minerals as well as multiple mineral hosts for a given element, the correlation between composite-based characteristics may confound the results of certain models. Despite not considering the effect of collinearity, the studies by Shangguan et al. (2022) and Trechera et al. (2021a) further described a positive correlation between siderite (iron-bearing carbonate) and oxidative potential, further affirming the contribution of iron-bearing minerals to the iron available for reaction (shown by Sun et al. (2021)). In a study by Song et al. (2022), the issue of collinearity, when relating composition-based data to inflammatory and cytotoxic responses, was mitigated using principal component analysis. By resolving multiple indicators of toxicity into principal components, a correlation matrix could be constructed using the composition data and the principal components for macrophages, epithelial, and fibroblasts cells. While this approach does not allow for the relative weighting of the characteristics that could be achieved in a multilinear model, it does allow for a preliminary screening of potential predictors and the interrelationships between them.

In both modelling applications, the analysis conducted only looked at associations between few characteristics and not the synergistic and antagonistic relationships between the marker of toxicity/inflammation and physicochemical and mineralogical characteristics of the dust. As a result, whilst additional chemical components were highlighted through correlation (such as potassium and titanium), no causative mechanism was linked to these components. Furthermore, these studies have not considered physical characteristics such as surface and shape when defining the pneumoconiotic potency of their coal mine dust samples, despite these parameters having been shown to greatly influence the functioning of phagocytes. Based on these limitations, it can be argued that a deeper investigation into statistical methods is needed to find ways of holistically defining the pneumoconiotic potency of sampled coal mine dust.

## Conclusions

Currently, CWP remains a prevalent occupational dust disease across the globe. In the context of the CWP resurgence in the USA, recent research suggests that the trends in coal mining in addition to the evolution in mining technologies employed have driven the changes in coal mine dust characteristics. Apart from the occupational setting, coal mine dust-related diseases have been documented in communities proximal to collieries (Laney & Weissman, 2014; Yadav & Jamal, 2018). Although extensive epidemiological surveys have been conducted to manage the burden of CWP and coal mine dust-related diseases in high-income countries, the latency of disease presents a challenge when attempting to connect current exposures and the future health outcomes. This implies that future migration strategies cannot solely rely on epidemiology, especially considering the advancements in tools used to understand the sources and characteristics of coal mine dust. Even so, with decades of research dedicated to understanding the pathology of CWP and the elucidation of causal factors related to its development, little consensus has been reached on the characteristics of coal which are responsible for disease development. Thus, it remains unclear which factor(s) should be taken forward in considering the pneumoconiotic potency of coals and, by extension, what actions need to be taken to mitigate the health effects caused from chronic coal mine dust exposure.

Generally, research efforts which have attempted to characterise coal mine dust and determine its source have identified that geo-anthropogenic alterations, resulting from crushing and wall rock contamination, may potentially impact the physicochemical properties of coal mine dust. By extension, it has been shown that these alterations may further impact the ROS generation potential of dust produced from different activities. As numerous studies have shown that pathologies resulting from coal mine dust are strongly mediated by cycles of ROS production and inflammation, characterising and understanding the anthropogenic alterations caused by mining and beneficiation processes may be important to mitigate the health effects. This is exemplified by the identification of silica-related r-type opacities within the lungs of miners who were exposed to dust contaminated by wall rock cutting.

Particle characteristics such as surface area/reactivity, particle shape, composition, and geochemistry have all been linked to the direct and/or indirect production of ROS. However, in the context of coal mine dust, mechanistic studies correlating particle characteristics to cytotoxicity and inflammation have mainly focused on composition-based data and have neglected the potential impact of surface area/reactivity and particle shape on macrophage functioning. In the context of pulmonary damage, both composition-related damage and morphology-driven macrophage impairment/stress are anticipated to occur simultaneously. Thus, research which can systematically define the relative contribution and magnitude of the coal particles’ physical, chemical, and mineralogical aspects to markers of respiratory harm could potentially assist in representing the pneumoconiotic potency of coal mine dust. Moreover, the development of screening methodologies for these various parameters would additionally assist in the evolution of targeted strategies to mitigate the variable pneumoconiotic potency of coal mine dust produced from different sources.
